# Pedobarographic Analysis following Ponseti Treatment for Unilateral Neglected Congenital Clubfoot

**DOI:** 10.1038/s41598-018-24737-w

**Published:** 2018-04-19

**Authors:** Chao Xu, Jie Wei, Ya-Bo Yan, Lei Shang, Xiao-Jiang Yang, Lu-Yu Huang, Wei Lei

**Affiliations:** 1 Department of Orthopedics, Xijing Hospital, Fourth Military Medical University, Xi’an, Shaan Xi Province China; 2 Department of Pathology, Xijing Hospital, Fourth Military Medical University, Xi’an, Shaan Xi Province China; 3 Department of Health Statistics, Faculty of Preventive Medicine, Fourth Military Medical University, Xi’an, Shaan Xi Province China

## Abstract

Recent trends have led to an interest in Ponseti treatment for correcting neglected congenital clubfoot. Although good clinical and functional outcomes have been reported, the plantar pressure distribution after the treatment of neglected clubfoot has not been explored yet. The present study aimed to investigate whether plantar pressures normalized following Ponseti treatment in patients with neglected congenital clubfoot. Pedobarographic, clinical, and functional examinations were performed in 22 children (aged, 91.0 ± 40.3 months) with unilateral neglected congenital clubfeet, treated using Ponseti method at 27.8 ± 12.1 months of age. Plantar pressure parameters were recorded using a Footscan pressure plate. The contact time, contact area, peak pressure, and pressure-time integral were determined. The data of the affected feet were compared with those of the unaffected feet and healthy controls. Although clinical and functional examinations showed satisfactory results according to the Dimeglio and Pirani scores, considerable differences in plantar pressure parameters were identified among the affected feet, unaffected feet, and healthy controls. Internal foot progression angle and a load transfer from the medial forefoot and hindfoot to the lateral forefoot and midfoot were observed in the affected feet. Future studies should attempt to investigate the factors accounting for plantar pressure deviations and the possible effect of these deviations on the lower limb musculoskeletal development of children.

## Introduction

Clubfoot is one of the most common congenital deformities of the lower extremity with an incidence of 0.6–0.8 per 1000 live births^1^. The deformity consists of hindfoot varus, forefoot adductus, equinus at the ankle, and cavus, which seriously affect the appearance and function of the foot. By definition, the neglected congenital clubfoot (NCF) is a foot that has not received any treatment by the time the child starts to walk^2^. In developing countries, NCF is prevalent owing to the lack of awareness and limited financial and social resources for health service^3^. The initial deformities of clubfoot are worsened by secondary changes due to weight-bearing and walking of the children as the side or dorsum of the affected foot begins to bear the weight, causing local skin ulcers, callosities and bursae formation, medial soft tissue contracture, and plastic deformation of bones^4^. The NCF can impose a heavy physical, psychological, social, and economic burden on the patients, families, communities, and countries^5,6^.

The main treatment of NCF is surgery, which consists of an extensive soft tissue release, tibialis anterior tendon transfer, different bony osteotomies, or even different types of external fixators^7–10^. These procedures are difficult, time-consuming, and costly. More importantly, the surgeries are often complicated by deep scarring, stiffness, and high recurrence rates^11–13^. Hence, many surgeons have adopted the minimally invasive Ponseti treatment for correcting NCF, which is the standard of care for treating patients presenting with clubfoot at younger than 1 year of age^14^.

In Brazil, Lourenco *et al*. applied Ponseti treatment in 17 patients with clubfoot (24 feet) between the age of 1.2 and 9 years. A painless, plantigrade foot was achieved in 66% of patients without further surgery^15^. In Nepal, Spiegel *et al*. reported the use of Ponseti treatment in 171 patients (260 feet) between the age of 1 and 6 years. Initial correction of the deformities was achieved, and extensive soft tissue release was avoided in 94% of patients^16^. In India, Verma *et al*. evaluated Ponseti treatment in 37 children (55 feet) between the age of 1 and 3years. Satisfactory outcomes were achieved in 89% of patients, which were painless, supple, plantigrade, and cosmetically acceptable feet^17^. Also, in India, Khan *et al*. treated 21 patients (25 feet) between the age of 7.5 and 11.1 years using the Ponseti method and achieved satisfactory results in 85.7% of patients^18^.

In the aforementioned studies, the treatment outcomes were assessed by clinical and functional examinations using scores and questionnaires. The examinations primarily focused on the range of foot motion in different planes, esthetics, muscle condition, pain relief, palpation result, bone realignment, or patient and family satisfaction. However, these assessments have some limitations: first, they consider the foot in a static state; second, they are difficult to quantify; and third, they suffer a certain degree of subjectivity^1,19^. They are not objective and comprehensive enough when the clinicians want the dynamic information of the foot, which is one of the most important aspects in evaluating the intervention outcomes^20,21^.

Pedobarographic analysis alleviates this problem and has proved to be a reliable clinical tool for quantifying the dynamic foot plantar pressure^22^. It can provide detailed temporal and loading information in various foot regions during the rollover process. Historically, the pedobarographic analysis has been shown to be useful in studying the gait characteristics of the patients with clubfoot after surgical or nonsurgical treatment^20,21,23–32^. However, to the best of our knowledge, no study to date has investigated the plantar pressures after Ponseti treatment for patients with NCF. Thus, the present study aimed to investigate whether plantar pressures normalized following Ponseti treatment in patients with NCF.

## Methods

### Subjects

Medical records were retrospectively reviewed to identify children who underwent Ponseti treatment for unilateral congenital clubfoot after the age of 1 year. Clinically asymptomatic children who had plantigrade feet and a minimum follow-up period of 12 months were included. The exclusion criteria included patients who were previously treated for clubfoot outside the department of orthopedics of Xijing Hospital, patients who needed orthotics to walk, and any patient who anticipated surgical management after the Ponseti treatment. The age-, sex-, height-, and weight-matched healthy children were recruited as the control group. This study was approved by the Ethical Committee of the Fourth Military Medical University. All experiments were performed in accordance with the Declaration of Helsinki. Written informed consent was obtained from all participants or their guardians before the commencement of the study.

### Treatment protocol

The Ponseti treatment was described in detail to the family at the initial clinical visit. Patients underwent weekly manipulation and application of corrective long leg casts in the outpatient operating room adhering to the Ponseti treatment^14^. Before each cast, manipulations lasted 5 min and were performed by a senior author experienced in the Ponseti method (Y.B.Y). Gentle manipulation of the feet was emphasized to avoid the complication of swollen feet. Then, a cast was applied for correcting cavus, forefoot adduction, and varus simultaneously. Prior to the last cast, a percutaneous tendoachilles tenotomy was performed in feet with persistent equinus. Then, a long leg cast was applied for 3 weeks in patients aged <3 years and for 4 weeks in patients aged >3 years^33^. Subsequently, foot abduction brace, which consisted of a Dennis Browne bar and straight-end shoes, was given to all the patients to prevent recurrence. The shoes were turned to 70° of external rotation for the affected foot and 45° of external rotations for the unaffected foot. It was worn by the patients for 23 h a day for the first 3 months and then only at a sleeping time until the age of 4–5 years. No special footwear was given to the patients for daytime to avoid any psychological stigmata. In the postcorrective protocol, gentle passive stretching exercise with the fulcrum at the hollow on the lateral aspect of the ankle over the site of unreduced talar head was extremely important^34^. The parents of the patients were instructed to perform the ankle stretching process four times a day for about 5 min each time.

### Anthropometric, clinical, and functional assessments

The present study evaluated the age of the patient at first casting, severity of deformity by the Dimeglio^35^ and Pirani^36^ scores prior to the Ponseti treatment, and at the last follow-up, the number of casts required for correction, and amount of ankle dorsiflexion at the last follow-up. The Dimeglio and Pirani scoring was done by the first author (C.X), which was rechecked by another author (L.Y.H).

### Instrumentation and pedobarographic analysis

The measurements of the foot pressure were collected from the healthy controls and all the patients at the last follow-up. Participants were required to perform pedobarographic tests barefoot at their self-selected walking pace. Plantar pressure data were recorded using a Footscan pressure plate (RSscan International, Olen, Belgium, 2096 × 472 × 18 mm^3^, with 16,384 resistive sensors arranged in a 256 × 64 matrix and a pressure range of 0–200 N/cm^2^). Each test comprised a minimum of three representative and reliable trials for each foot. Each participant was required to take a 3-min break every 10 min to prevent fatigue.

The Footscan platform was located at the center of a carpet with the same external dimension to provide a “complete platform”, 4 m in length, to ensure that a minimum of three steps were taken before data collection^37^. Before collecting the dynamic data, all the participants completed 15-min acclimatization walking trails along the measuring platform.

**Figure 1:**
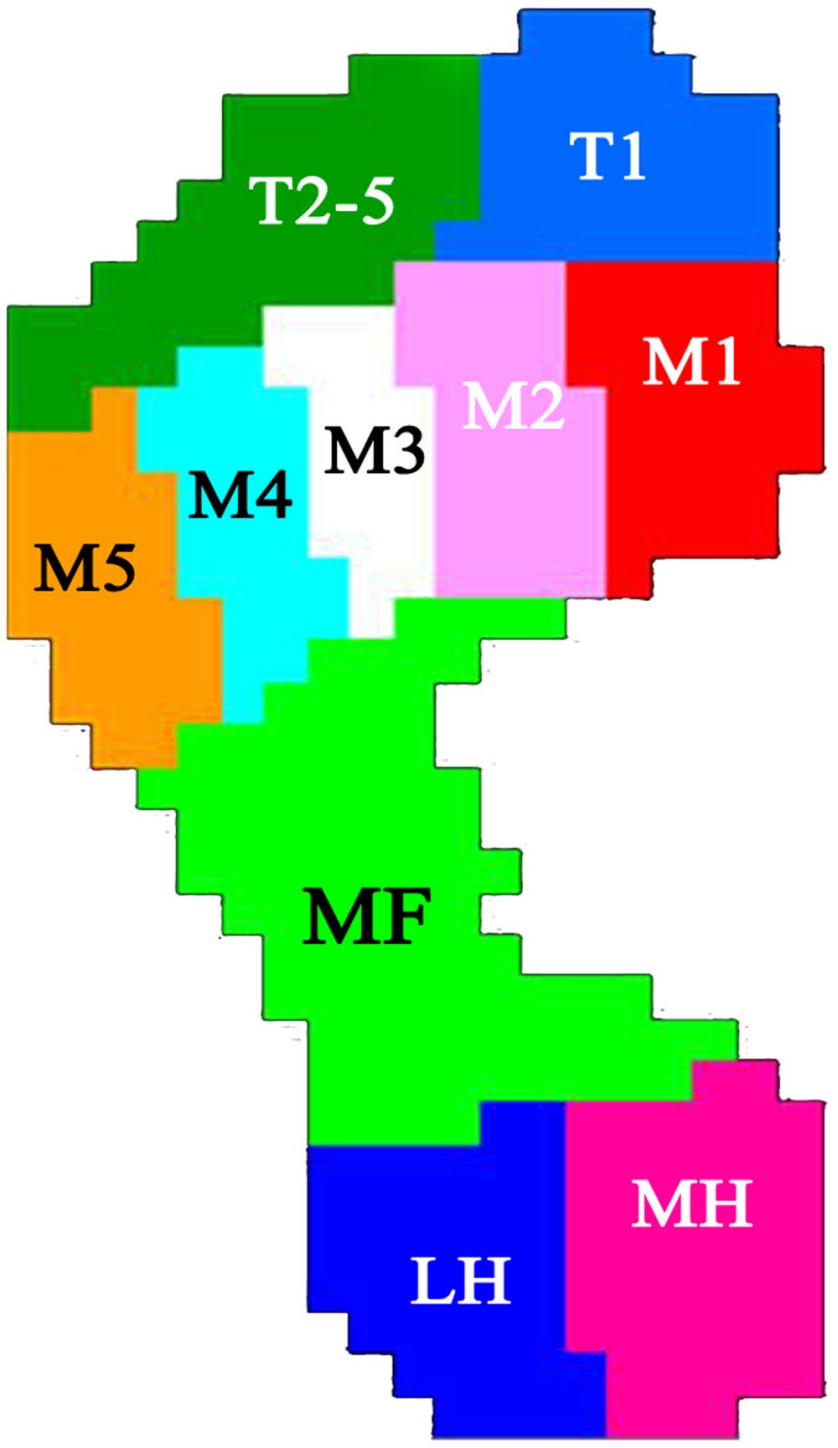
Schematic diagram for the 10 subdivided zones of the foot. The subdivided zones were (T1) hallux, (T2–5) toes 2–5, (M1) first metatarsal, (M2) second metatarsal, (M3) third metatarsal, (M4) fourth metatarsal, (M5) fifth metatarsal, (MF) midfoot, (MH) medial heel, and (LH) lateral heel.

The data were analyzed using Scientific Footscan software (RSscan International, Olen, Belgium), which automatically split the foot into 10 masked zones: medial hindfoot (MH), lateral hindfoot (LH), midfoot (MF), first to fifth metatarsals (M1, M2, M3, M4, and M5), hallux (T1), and toes 2–5 (T2–5) (Fig. 1). After each measurement, a visual checking and manual correction were made by the same experienced observer (C.X) to assure that the anatomical structures fitted with the masked zones^26,38^.

**Figure 2:**
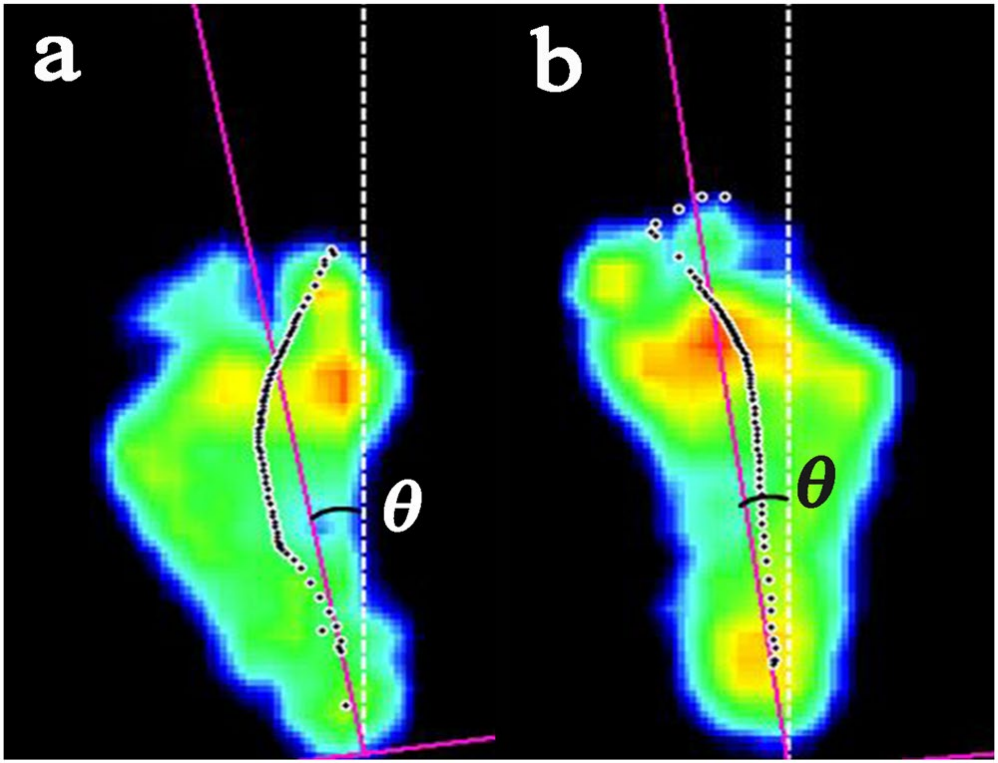
Examples of the foot progression angle. The purple line is located between the medial and lateral parts of the heel and between the second and third metatarsal heads. The white dotted line is the gait direction. (**a**) Exo rotation of the left foot (*θ* = 13.36°). (**b**) Endo rotation of the right foot (*θ* = −10.53°).

The outcome variables quantifying the plantar pressure for each masked zone included the following: contact time reported as a percentage of the stance time (CT%); contact area reported as a percentage of the total foot area (CA%); peak pressure (PP, kPa); and pressure-time integral (PTI, kPa s). The foot progression angle (FPA) was calculated as the angle between the gait direction and the line located between the medial and lateral parts of the heel and between the second and third metatarsal heads^39^. A positive angle indicated exo rotation of the foot, whereas a negative angle indicated endo rotation of the foot (Fig. 2a,b). For the Ponseti group, the values recorded for each parameter were the means of the three representative trials. For the control group, each variable was calculated by averaging the values across the feet^40^.

### Statistical analysis

Statistical analysis was performed using SPSS, version 19.0 software (SPSS Inc., IL, USA). The data were explored for outliers and distribution. Normality was investigated using the one-sample Kolmogorov-Smirnov test. Normally distributed data were presented as mean ± standard deviation. A *P* value < 0.05 was used throughout as an indicator of statistical significance. Differences in the plantar pressure parameters of interest between the affected and unaffected feet of the Ponseti group were determined using the paired *t* tests. Differences between the Ponseti group and the controls were determined using the independent sample *t* tests^30^. Pretreatment and posttreatment Dimeglio scores were compared using the paired *t* tests. Pretreatment and posttreatment Pirani scores were also compared using the paired *t* tests. The chi-square test was used to compare the rates of internal FPA among different groups.

## Results

A total of 22 patients with unilateral NCF (15 boys and 7 girls) who underwent Ponseti treatment comprised the Ponseti group. The left foot was affected in 11 patients and the right in 11 patients. No patient was lost to follow-up. The average age of the patients at the first cast was 27.8 ± 12.1 months, and the mean follow-up time was 63.1 ± 41.4 months. Similarly, 22 healthy children (15 boys and 7 girls) were selected as the control group. No significant differences were observed in age, body mass, and height between the two groups (Table 1).

**Table 1:** Demographic characteristics of the study cohort by group.

	Ponseti group (*n* = 22)	Control group (*n* = 22)	*P*
Age at Ponseti treatment (month)	27.8 ± 12.1		
Follow-up (month)	63.1 ± 41.4		
Age at pedobarographic analysis (month)	91.0 ± 40.3	95.8 ± 55.5	0.741
Body mass (kg)	27.3 ± 12.1	28.4 ± 14.3	0.786
Body height (cm)	126.9 ± 20.7	124.4 ± 27.0	0.737

Values are expressed as means ± standard deviation.

The mean Dimeglio score before treatment and at the last follow-up was 13.5 ± 2.3 and 1.1 ± 0.9, respectively. The mean Pirani score before treatment and at the last follow-up was 4.9 ± 0.8 and 1.0 ± 0.5, respectively. Both the scores improved significantly after the Ponseti treatment (*P* < 0.001). All patients underwent percutaneous tendoachilles tenotomies. All the feet achieved full correction with a mean of 11.3 ± 1.9 casts. The average dorsiflexion achieved was 14.2° ± 4.4° at the last follow-up. All the patients could wear normal shoes and walk on plantigrade feet.

The mean values and standard deviations of the CT% in the 10 masked zones and the total CT in different groups are shown in Table 2. The mean values of CT% significantly increased in the M5 and MF zones and significantly decreased in the M1 and M2 zones in the affected feet compared with those in the control group. In the unaffected feet, the values of CT% were higher in the MF, MH, and LH zones and lower in the T1 zone compared with those in the control group. In the Ponseti group, lower values of CT% in the M2 and higher values in the M5 and MF zones were recorded in the affected feet than in the unaffected side. In terms of total CT, the mean value in the affected feet was significantly smaller than that in the unaffected side and control group.

**Table 2:** Comparison of the CT% in the 10 masked zones and total CT (millisecond) in different groups.

	Ponseti group		Control	*P*g		*P*s
	A	U		A	U	
T1	41.1 ± 22.6	35.3 ± 11.0	48.0 ± 11.9	0.213	0.001^*^	0.320
T2–5	44.0 ± 31.8	34.8 ± 27.7	33.4 ± 16.1	0.173	0.843	0.275
M1	55.1 ± 26.3	61.4 ± 22.7	69.1 ± 14.3	0.034^*^	0.185	0.322
M2	56.6 ± 23.6	67.7 ± 18.7	68.8 ± 12.2	0.037^*^	0.827	0.022^*^
M3	64.6 ± 20.8	70.0 ± 17.2	70.8 ± 11.3	0.232	0.873	0.116
M4	69.7 ± 20.2	63.6 ± 20.6	67.1 ± 12.1	0.600	0.499	0.142
M5	66.5 ± 22.6	48.3 ± 23.8	54.3 ± 15.6	0.045^*^	0.325	0.001^*^
MF	73.2 ± 11.8	62.8 ± 11.5	53.5 ± 9.8	<0.001^*^	0.007^*^	0.001^*^
MH	56.3 ± 17.2	58.2 ± 10.7	50.2 ± 12.5	0.188	0.028^*^	0.542
LH	55.4 ± 17.0	57.3 ± 10.7	48.7 ± 11.9	0.136	0.015^*^	0.548
Total CT	564.7 ± 100.9	625.0 ± 94.0	620.1 ± 76.1	0.046^*^	0.850	<0.001^*^

^*^P < 0.05.

Values are expressed as means ± standard deviation.

*P*g: comparisons between the Ponseti group and the control group using independent sample *t* test; *P*s: comparisons between the two feet in the Ponseti group using paired *t* test.

CT = contact time, CT% = contact time reported as a percentage of the stance time, A = affected feet in the Ponseti group, U = unaffected feet in the Ponseti group, T1 = hallux, T2-5 = toes 2–5, M1 = first metatarsal, M2 = second metatarsal, M3 = third metatarsal, M4 = fourth metatarsal, M5 = fifth metatarsal, MF = midfoot, MH = medial heel, LH = lateral heel.

The total CA in the affected feet was smaller than those in the unaffected feet and control group. Meanwhile, the affected feet showed significantly increased CA% in the M5 and MF zones and decreased CA% in the M1 and MH zones compared with values in the unaffected feet and control group. The differences between the unaffected feet and the control group were not statistically significant (Table 3).

**Table 3:** Comparison of the CA% in the 10 masked zones and total CA (cm^2^) in different groups.

	Ponseti group		Control	*P*g		*P*s
	A	U		A	U	
T1	8.9 ± 2.4	9.5 ± 2.1	9.8 ± 1.7	0.133	0.523	0.323
T2–5	7.8 ± 3.3	8.0 ± 3.3	8.9 ± 2.1	0.180	0.251	0.814
M1	6.0 ± 2.5	8.9 ± 2.6	7.7 ± 1.5	0.009^*^	0.075	0.001^*^
M2	6.9 ± 1.1	7.1 ± 1.5	7.1 ± 1.0	0.502	0.991	0.586
M3	6.0 ± 1.0	5.8 ± 1.2	5.6 ± 0.7	0.142	0.619	0.515
M4	6.7 ± 1.5	6.1 ± 1.1	6.5 ± 0.7	0.655	0.097	0.101
M5	8.7 ± 2.7	6.9 ± 2.0	7.3 ± 1.4	0.042^*^	0.424	0.023^*^
MF	30.5 ± 7.0	26.5 ± 4.5	26.3 ± 3.5	0.017^*^	0.897	0.005^*^
MH	9.8 ± 2.8	11.7 ± 1.7	11.2 ± 1.3	0.038^*^	0.332	0.009^*^
LH	8.8 ± 2.2	9.7 ± 1.4	9.4 ± 1.2	0.251	0.459	0.086
Total CA	91.6 ± 22.5	105.1 ± 22.0	107.3 ± 26.3	0.040^*^	0.771	<0.001^*^

^*^P < 0.05.

Values are expressed as means ± standard deviation.

*P*g: comparisons between the Ponseti group and the control group using independent sample *t* test; *P*s: comparisons between the two feet in the Ponseti group using paired *t* test.

CA = contact area, CA% = contact area reported as a percentage of the total foot area, A = affected feet in the Ponseti group, U = unaffected feet in the Ponseti group, T1 = hallux, T2-5 = toes 2–5, M1 = first metatarsal, M2 = second metatarsal, M3 = third metatarsal, M4 = fourth metatarsal, M5 = fifth metatarsal, MF = midfoot, MH = medial heel, LH = lateral heel.

**Figure 3:**
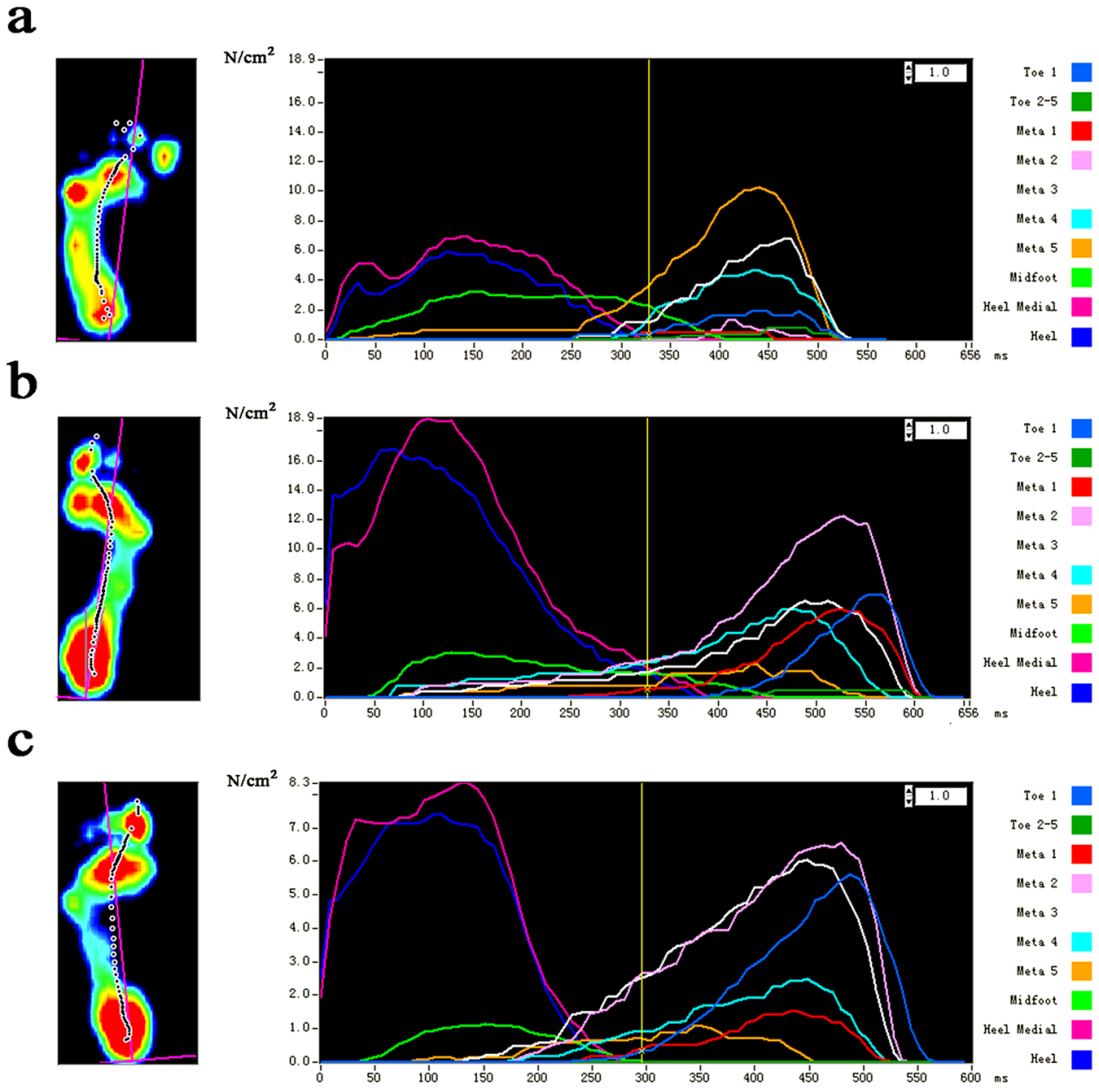
The curves of the peak pressure (PP) for the 10 masked zones of a representative subject. (**a**) Curves of the PP in the affected foot. (**b**) Curves of the PP in the unaffected foot. (**c**) Curves of the PP in the control foot. The subdivided zones were (Toe 1) hallux, (Toe 2–5) toes 2–5, (Meta 1) first metatarsal, (Meta 2) second metatarsal, (Meta 3) third metatarsal, (Meta 4) fourth metatarsal, (Meta 5) fifth metatarsal, (Midfoot) midfoot, (Heal medial) medial heel, and (Heel) lateral heel. 1 N/cm^2^ = 10 kPa.

Table 4 summarizes the results of PP in different groups. Compared with the controls and the unaffected side, the affected feet showed a higher PP in the M5 and MF zones and a lower PP in the T1, M1, M2, MH, and LH zones (Fig. 3). The differences between the unaffected feet and the control group were not statistically significant.

**Table 4:** Comparison of the PP (kPa) in the 10 masked zones in different groups.

	Ponseti group		Control	*P*g		*P*s
	A	U		A	U	
T1	32.0 ± 12.6	40.7 ± 15.6	50.3 ± 22.9	0.002^*^	0.111	0.002^*^
T2–5	8.3 ± 4.2	6.8 ± 3.6	8.5 ± 4.0	0.884	0.139	0.222
M1	31.2 ± 12.4	49.2 ± 18.9	40.2 ± 14.7	0.034^*^	0.085	<0.001^*^
M2	61.2 ± 25.5	84.5 ± 26.6	84.0 ± 26.8	0.006^*^	0.955	0.007^*^
M3	84.7 ± 26.6	91.3 ± 29.2	95.0 ± 20.7	0.162	0.636	0.240
M4	67.6 ± 18.2	60.6 ± 16.6	62.0 ± 17.8	0.308	0.791	0.177
M5	47.0 ± 18.8	25.1 ± 10.7	27.4 ± 9.2	<0.001^*^	0.450	<0.001^*^
MF	50.5 ± 19.7	20.4 ± 9.0	18.6 ± 6.2	<0.001^*^	0.433	<0.001^*^
MH	88.2 ± 24.7	120.3 ± 37.0	104.9 ± 29.8	0.049^*^	0.137	0.003^*^
LH	79.9 ± 21.7	91.8 ± 21.4	92.5 ± 18.6	0.044^*^	0.905	0.043^*^

^*^P < 0.05.

Values are expressed as means ± standard deviation.

*P*g: comparisons between the Ponseti group and the control group using independent sample *t* test; *P*s: comparisons between the two feet in the Ponseti group using paired *t* test.

PP = peak pressure, A = affected feet in the Ponseti group, U = unaffected feet in the Ponseti group, T1 = hallux, T2-5 = toes 2–5, M1 = first metatarsal, M2 = second metatarsal, M3 = third metatarsal, M4 = fourth metatarsal, M5 = fifth metatarsal, MF = midfoot, MH = medial heel, LH = lateral heel.

Table 5 shows the comparison of the PTI in different groups. The affected feet showed significantly increased PTI% in the M5 and MF zones and decreased PTI% in the T1, M1, M2, and MH zones compared with the control group. Meanwhile, lower PTI in the T1 and T2–5 zones were noted in the unaffected feet compared with corresponding values in the control group. In the Ponseti group, higher values of PTI in the M5 and MF zones and lower values in the M1, M2, and MH zones were noted in the affected feet than in the unaffected side.

**Table 5:** Comparison of the PTI (kPa s) in the 10 masked zones in different groups.

	Ponseti group		Control	*P*g		*P*s
	A	U		A	U	
T1	3.3 ± 1.7	4.5 ± 1.8	6.0 ± 2.9	<0.001^*^	0.040^*^	0.079
T2–5	1.3 ± 0.6	1.1 ± 0.3	1.4 ± 0.5	0.610	0.043^*^	0.213
M1	4.1 ± 2.2	8.0 ± 3.7	6.2 ± 2.5	0.005^*^	0.062	<0.001^*^
M2	10.9 ± 3.3	15.5 ± 5.6	14.8 ± 6.8	0.021^*^	0.675	0.001^*^
M3	15.1 ± 6.6	17.9 ± 6.8	19.0 ± 8.6	0.106	0.643	0.069
M4	15.3 ± 5.9	13.0 ± 4.9	13.4 ± 4.3	0.213	0.794	0.152
M5	9.5 ± 3.8	5.1 ± 2.9	6.4 ± 3.0	0.005^*^	0.137	0.002^*^
MF	11.7 ± 4.9	4.7 ± 1.9	3.7 ± 1.4	<0.001^*^	0.066	<0.001^*^
MH	16.8 ± 6.7	24.5 ± 8.0	22.2 ± 9.2	0.031^*^	0.371	<0.001^*^
LH	16.7 ± 7.1	18.3 ± 8.5	15.0 ± 7.0	0.441	0.168	0.446

^*^P < 0.05.

Values are expressed as means ± standard deviation.

*P*g: comparisons between the Ponseti group and the control group using independent sample *t* test; *P*s: comparisons between the two feet in the Ponseti group using paired *t* test.

PTI = pressure-time integral, A = affected feet in the Ponseti group, U = unaffected feet in the Ponseti group, T1 = hallux, T2-5 = toes 2–5, M1 = first metatarsal, M2 = second metatarsal, M3 = third metatarsal, M4 = fourth metatarsal, M5 = fifth metatarsal, MF = midfoot, MH = medial heel, LH = lateral heel.

In terms of the FPA, 63.6% (14/22) of the affected feet showed an internal FPA, while only 22.7% (5/22) of the unaffected feet were found to have an internal FPA; the value in the control group was 31.8% (7/22). The affected feet showed a higher proportion of internal FPA compared with the unaffected side (*P* = 0.006) and the control group (*P* = 0.035).

## Discussion

NCF is common in developing countries due to the lack of an appropriate health care system^6,41^. The deformity of NCF is complex, and hence the surgeons have not been able to treat it successfully. More importantly, the children with NCF deformity are unable to get into the mainstream of society because of physical disability and stigmatization^33^. In the present study, successful clinical outcomes were achieved in patients after the Ponseti treatment, defined as substantially improved Dimeglio and Pirani scores^16^. However, residual deviations in plantar pressures were still observed in these patients.

Some studies used the plantar pressure measurement systems to evaluate the outcome of patients presenting with clubfoot at an early age following nonoperative and surgical management. They found that the clubfeet treated using Ponseti method had higher PP and PTI in the midfoot^26,28–30^ and lateral forefoot^26,30^, whereas the PP and PTI were substantially lower in the medial forefoot^26,28–30^ and hindfoot^26,29,30^, compared with the healthy controls. Similarly, in the present study, the PP and PTI were higher in the M5 and MF zones and lower in the affected feet in the M1, M2, and MH zones compared with those in the unaffected side and control group. These results indicated a load transfer from the medial forefoot and hindfoot to the lateral forefoot and midfoot^24,28^. The deviations could be attributed to the more medially progression angle^26,42^, limitation in dorsiflexion of the affected foot^24,32^, lower ankle plantar flexion moment due to calf weakness^1,43^, midfoot break appearance, and more lateral position of the foot in relation to the ground^26^. These changes might be a sign of under correction or recurrence^29^ and are difficult to detect by clinical examination. Cooper *et al*.^32^ suggested a certain tolerance toward the shift of the pressure because no substantial effect on the overall function was found in their study. However, Jean *et al*. were concerned that the high loading on the lateral side of the foot might eventually lead to skin problems, pain^26^, and lower extremity overuse injuries^44^. A longer follow-up is needed to monitor and assess the effect of the high loading on the lateral part of the affected clubfeet.

In the present study, a higher proportion of the internal FPA was found in the affected clubfeet than in the unaffected side and control group. Similarly, other studies reported the “toe-in” gait pattern in patients with clubfoot following nonoperative and surgical treatments^24,27,28,43,45^. The change could be attributed to the increased tibialis anterior muscle activity^46^, residual forefoot and hindfoot deformities^47^, reduced external tibial or femoral torsion^23^, persistent medial hindfoot spin^48^, and talocalcaneal malaligment^49^. Some studies reported that the internally rotated FPA shifted the loading to the lateral side of the foot^26,42^, which was also consistent with the findings of the present study.

Similar to previous reports^26,28,30^, the present study showed that the total CA of the affected feet was smaller than those of the unaffected feet and control group. Therefore, using the absolute value of CA could cause a bias when comparing CA in a clubfoot with that in a healthy foot^28^. Consequently, CA was normalized to the total foot area (CA%) in this study. The affected feet showed a significant increase in CA% in the M5 and MF zones compared with the controls and the unaffected feet. Meanwhile, a higher PP was found in the M5 and MF zones in the affected clubfeet compared with the values in the other two groups. Theoretically, plantar pressures are the result of the vertical ground reaction force (GRF) exerted on the foot during the rollover process divided by the CA. Therefore, the increased CA and PP demonstrated a substantially elevated vertical GRF in the affected feet of the patients with NCF. It suggested that the affected feet experienced substantially higher mechanical stresses during walking. However, PP represented only the vertical component of the applied tissue stress, and shear forces were also crucial in plantar ulcer formation^50,51^. Future studies should focus on investigating shear pressures, especially in the sites of higher PP, thereby providing more information regarding tissue stresses.

The unaffected feet of patients with unilateral clubfoot were selected as controls in some studies on plantar pressure. However, a few studies reported considerable differences in foot loading parameters between the unaffected feet and healthy controls^20,30,52^. The present study found that the CT% and PTI in the unaffected feet were substantially different from the control values. These differences could be attributed to the residual gait deviations on the affected side. The alterations in the affected side could induce an asymmetrical gait pattern reflected on the unaffected side. The central nervous system might adapt its control on the unaffected side to preserve a global symmetry of the gait^20,52^. Finally, the plantar pressure distributions of the unaffected feet changed. These results indicated that, in addition to the affected feet, the unaffected feet also needed attention during the follow-up period.

This study had some limitations. First, the association between the clinical outcomes and plantar pressure parameters of the patients with NCF could not be investigated because all patients showed satisfactory clinical results due to the inclusion criteria of the study. However, these feet still showed considerable differences in plantar pressure distribution. Further studies including patients with worse clinical outcomes might facilitate exploring a relationship between the outcome evaluations and foot loading parameters. Second, the follow-up time was short and the long-term results of the treatment in the present study were still unknown. Therefore, additional follow-up was necessary to identify further changes in presentation in the teenage years and into adulthood. Third, the selection of a representative trial and manual corrections to the masked zones were subjective and a limitation of the study. In addition, collecting the qualified plantar pressure data in young children was difficult, especially in a patient after treatment. In the event that a child was unable to complete the pedobarographic testing, toys, candies, or parental guidances was used to obtain their cooperation. These factors might have affected the quality of the data. However, the researchers involved in the present study were experienced in testing young children, and other studies collected qualified plantar pressure data using similar experimental protocols in young children^27,28,30^.

This study reported a pedobarographic analysis of 22 children with unilateral NCF deformity at an average of 63 months after Ponseti treatment. Although the clinical and functional outcomes after Ponseti treatment were encouraging, plantar pressure deviations were found in both the affected and unaffected feet. The deviations might be attributed to some level of under correction or recurrence, residual lower limb musculoskeletal deformity, and adaptations of the central nervous system. A longer follow-up was needed to evaluate the possible effect of these deviations on the lower limb musculoskeletal development of children, even if they recovered a normal life. Future studies should attempt to investigate the factors accounting for plantar pressure deviations and the relationship between the clinical outcomes and plantar pressure parameters.
